# Deep brain stimulation for arm tremor: one-year blinded follow-up of a randomized trial

**DOI:** 10.1186/s42466-026-00537-w

**Published:** 2026-10-09

**Authors:** Nadja Kvernmo, Martin M. Reich, Jonas Roothans, Ane E. Konglund, Are H. Pripp, Espen Dietrichs, Jens Volkmann, Inger Marie Skogseid

**Affiliations:** 1 Department of Neurology, Oslo University Hospital, PO box 4950, Nydalen, Oslo, 0372 Norway; 2 Institute of Clinical Medicine, University of Oslo, Oslo, Norway; 3 Department of Neurology, University Hospital of Würzburg, Würzburg, Germany; 4 Visual DBS lab, University Hospital of Würzburg, Würzburg, Germany; 5 Department of Neurosurgery, Oslo University Hospital, Oslo, Norway; 6 Department of Biostatistics, Oslo University Hospital, Oslo, Norway; 7 Institute of Clinical Medicine, University of Würzburg, Würzburg, Germany

## Abstract

**Objective:**

To present the secondary endpoints at one-year blinded follow-up from our randomized controlled study, that compared the efficacy of DBS in the ventral-intermediate thalamic nucleus (VIM) with the posterior subthalamic area (PSA) three months each in crossover design, and showed significantly greater reduction of the Sum dominant arm tremor score (Fahn-Tolosa-Marin) during the PSA-DBS period. The outcomes at one year are now reported to verify target choice and sustained benefit with clinically optimized free programming.

**Methods:**

Forty-five patients with isolated or combined arm action tremor received four-level DBS leads to reach both VIM (upper two levels) and PSA (lower two), and were randomized post-operatively to VIM-DBS month 1–3 followed by PSA-DBS month 4–6, or vice versa. Thereafter electrode choice was free as judged by best clinical outcome. Predefined secondary endpoints were the tremor score improvements from preoperative to the one-year follow-up for dominant and non-dominant arm, other body regions, total score, two patient-rated measures and adverse events.

**Results:**

At the one-year follow-up, Sum dominant arm tremor scores had improved from median 24 at baseline to 4 *(p*<.001, *n* = 43), corresponding to median 79% improvement. All other secondary endpoints also improved significantly (*p*<.001). Median action tremor improvement was for Essential tremor(ET) 83%, for Parkinson´s disease(PD) and Dystonic tremor 100% and for Cerebellar tremor 55%. Over 90% of stimulated contacts were in PSA.

**Interpretation:**

At one year, good and sustained tremor suppression from PSA-DBS was confirmed in ET, PD and Dystonic tremor. Suppression of Cerebellar tremor was more variable.

**Trial registration:**

NCT03156517, May 29, 2017.

**Supplementary Information:**

The online version contains supplementary material available at https://doi.org/10.1186/s42466-026-00537-w.

## Introduction

Action tremor of the arms is the most prominent symptom in Essential Tremor (ET), Dystonic Tremor, and Cerebellar Tremor, and occurs frequently together with rest tremor in Parkinson´s disease (PD). With increasing severity it affects many daily functions. Oral medications such as propranolol, primidone, and topiramate for ET and levodopa for PD often provide insufficient tremor suppression, and for cerebellar and dystonic tremor no class-1 evidence medication can be offered, although benzodiazepines and other agents modulating GABA may provide some relief. Moreover, pharmacotherapy for tremor is often limited by intolerable side effects [1, 2].

For the past three decades, deep brain stimulation (DBS) of the thalamic ventral intermediate nucleus (VIM) has been established as an effective treatment for patients with medication-refractory tremor in ET and PD, both in the short and long term [3–8]. Patient satisfaction with VIM-DBS has, however, been limited by a relatively high frequency of stimulation-induced adverse effects such as paresthesias, dysarthria and gait ataxia, especially with bilateral stimulation, and by development of tolerance or habituation [3–8]. Tolerance is the phenomenon that after a variable duration of stable, good tremor suppression from DBS, the effect wanes over time and can only temporarily be improved by increasing stimulation amplitude, pulse width or frequency. It may occur after months to years and has been reported in varying frequency, from 0 to 73% [9]. The causes are debated, with the most convincing hypotheses being underlying disease progression, particularly of cerebellar pathology, and/or stimulation-induced plastic changes in the cerebello-thalamic networks involved in tremor suppression [9].

In the search for a better target, several early patient series showed promising results of stimulation just below VIM, in the Posterior Subthalamic Area (PSA) [10–13]. PSA consists of the Zona Incerta nucleus, the pallido-thalamic tract, and the prelemniscal radiation, which includes several myelinated cerebello-thalamic pathways, including the dentato-rubro-thalamic tract (DRTT). Data from a randomized controlled trial that compared PSA- and VIM-DBS as the primary endpoint and with sufficient power were, however, lacking until we published the results from the randomized phase (first six months) of our study in 2022, where we investigated the effect on action tremor as a symptom, in a clinically heterogeneous patient cohort. By using one lead to reach both targets and a crossover design, we compared the intra-individual differences in efficacy of VIM- versus PSA-DBS for three months each, and found a significantly better effect of PSA-DBS during month 4–6 post-surgery, while the effect was similar during month 1–3 [14]. The latter we attributed to the surgical stun effect. Two large retrospective studies that compared localization of active contacts to the degree of tremor suppression were published simultaneously and concluded that stimulation in the PSA, in close proximity to the DRTT, was superior [15–17].

We here present the results from the double-blinded one-year follow-up of our cohort, after DBS had been optimized individually since the end of the six-month randomized phase, to confirm whether the effect on tremor suppression was sustained in all diagnostic categories and to verify the preferred target for chronic treatment.

## Methods

### Study design and participants

**Fig. 1:**
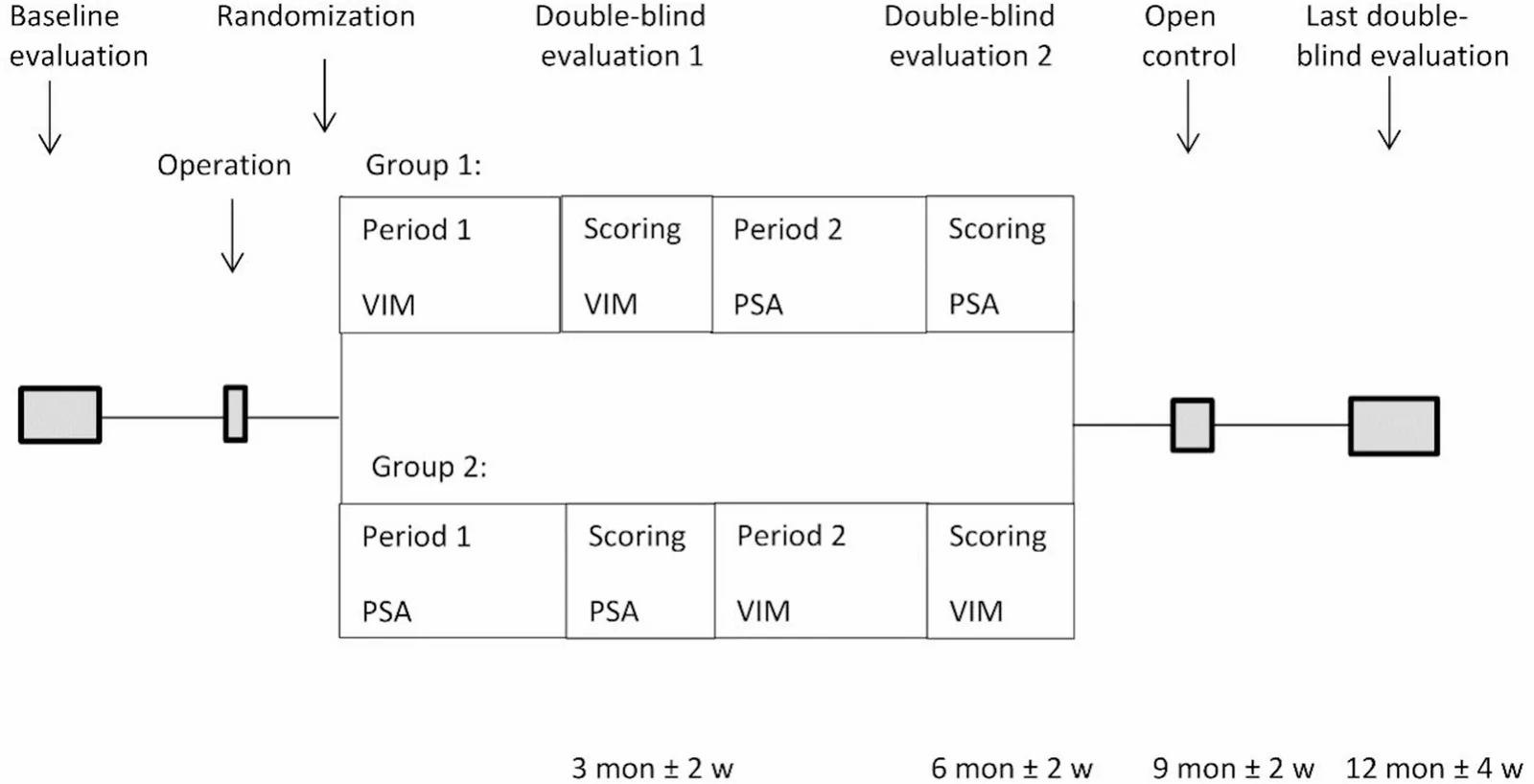
Study design

From April 2014 to December 2018, we included 45 patients in a single-center, randomized, double-blind, crossover trial that compared the efficacy of VIM-DBS to PSA-DBS on arm action tremor as a symptom of the most common tremor disorders: ET, PD, Dystonic tremor or Cerebellar tremor. Cerebellar tremor was defined as a clear intention tremor, with a crescendo of the tremor amplitude in Baranys test, plus at least one other cerebellar sign from that arm such as dysdiadochokinesia or ataxia. Four-level DBS leads were implanted (bi- or unilaterally as clinically indicated) with one trajectory to reach both VIM and PSA. Patients were randomized post-surgery to receive stimulation in VIM first then PSA, or opposite order, for three months each. After this six-month randomized phase, stimulator settings were optimized through clinical testing until the last double-blind evaluation of the predefined secondary endpoints was performed at one year post-surgery. Figure 1 summarizes the study design, which has been published in detail previously together with the results of the randomized phase [14].

In short, all patients were operated and followed at the DBS center, Oslo University Hospital (OUH), Oslo, Norway. The study protocol was approved by the Regional Committee for Medical Research Ethics of South-Eastern Norway (2013/1013) and the Personvernombud (Privacy Ombudsman), Oslo University Hospital before the intiation of the study. It was registered at Clinicaltrials.gov (NCT03156517, May 29, 2017). All patients gave their written informed consent to participate and consent to publication before inclusion. After inclusion and operation they were randomized (1:1 allocation) to receive one of two treatment sequences (Group 1:VIM-DBS first, then PSA-DBS (*n* = 23), Group 2:PSA-DBS first, then VIM-DBS (*n* = 22), through block randomization, using a computer-generated, secured, web-based service. Two dropouts occurred late in the second randomized period. A 57-year old woman with dystonic tremor developed stimulator-site infection that necessitated device removal, and a 58-year old man with cerebellar tremor secondary to advanced multiple sclerosis(MS) died of pneumonia (after 2.5 months of stable stimulation parameters). The remaining 43 patients continued in the study.

### Neurological evaluation and scoring

Preoperatively and three, six and 12 months post-surgery, tremor severity and disability were scored using the Fahn-Tolosa-Marin Tremor Rating Scale (FTMTRS) [18]. In patients with PD (*n* = 10), the Unified Parkinson´s Disease Rating Scale part III (motor examination) was also scored after an over-night withdrawal of dopaminergic medication and one hour after receiving 1.5 times their morning levodopa dose. All patients were interviewed using the Quality of life in Essential Tremor Questionnaire (QUEST) [19], scored for Global Burden of Disease (VAS-GBD) for tremor and for pain on a Visual Analog Scale, and evaluated for symptoms of anxiety and/or depression on the Hospital Anxiety and Depression Scale (HADS) [20] .

One physician (NK) performed the clinical evaluations at all study visits, including complete neurological examination preoperatively and at one-year follow-up, and systematic evaluation of adverse events (AEs) at all follow-ups using standardized forms to support focused clinical interview/examination. She was blinded to the contacts and stimulation parameters. Information on AEs was also monitored by the project leader (IMS) who programmed the stimulators. Patients were blinded to stimulation target the first year during which they had no access to the patient control device.

For description of the FTMTRS items [18] see the legend of Table 2. QUEST is a 30-item questionnaire developed and validated for ET patients, with five domains evaluating physical aspects, communication, work, hobbies/leisure, and psychosocial aspects of tremor [19]. A Summary Index is calculated (sum of the domain scores/5). On the VAS-GBD, patients evaluated disease burden by marking on a continuous 100 mm line (0 = no burden; 100 = maximum burden imaginable). HADS is a well-established, validated self-report questionnaire containing 14 items expressing emotions, alternating between seven emotions of anxiety and seven of depression (each item scored 0–4, higher scores indicate more emotional distress). We applied cut-offs for HADS Anxiety ≥10 and HADS Depression ≥9 for clinically significant anxiety and depression, respectively [20].

### Surgical procedure

For details refer to our previous publication. In short, preoperative 3-Tesla MRI sequences were merged with computed tomography scan (CT) after mounting a stereotactic head ring in parallel with the intercommissural line (ICL), using the iPlan™ (version 3.0 or later) computer-aided neuronavigation system (BrainLab, Germany). The targets were planned using predefined stereotactic coordinates (indirect targeting) for VIM and structural/anatomical guidance (direct targeting) for PSA. Our definition of the VIM target point was X = 50% of the ICL length lateral to the ICL, Y = 30% anterior to the posterior commissure, and Z=level of the ICL-plane. The PSA was targeted slightly posterior and medial to the subthalamic nucleus (STN), at the level of the maximal diameter of the red nucleus (RN), aligned with published coordinates. A trajectory angle was applied to allow the two most dorsal contact levels to reach the VIM and the two most ventral to reach the PSA. The electrodes were implanted in awake patients and test-stimulation performed from 2 mm above the AC-PC-level to 4 mm below, at 2 mm steps to observe tremor suppression and threshold for AEs [14].

### Modulation of stimulation parameters

During the randomized phase, we tested the two upper contacts of the lead (*N* = 38, Medtronic^®^ 3389, *N* = 7, Boston^®^ Cartesia) with monopolar review in the VIM-period and the two lowest in the PSA-period, chose the contact that yielded the best clinical results, and kept stimulator settings as similar as possible in the two randomized periods (monopolar, ring-stimulation mode, current ≤ 3 milliAmpere, pulse width 60 microseconds, frequency 130 Hz (< 145)). After the randomized six-month period, the project leader freely selected the contact yielding the best tremor reduction with no or only minor side effects at up to two clinical controls, to optimize stimulation parameters (in ring-stimulation mode) before the last double-blinded study evaluation at one-year follow-up.

### Outcomes

The primary endpoint of the randomized phase was the difference in reduction of Sum dominant arm tremor score (FTMTRS items 5/6 + 10–14) from baseline to the end of the VIM-DBS versus the PSA-DBS period, studying paired intra-individual differences (each patient serving as their own control) [14]. The pre-specified secondary endpoints presented here are the paired intra-individual differences from preoperative to the one-year blinded follow-up in the following scores: Sum dominant arm tremor (same items as primary endpoint), Sum non-dominant arm tremor (item 5/6 + 11–14), tremor scores for face/tongue/voice, head, trunk and lower limb(s), total FTMTRS score, QUEST Summary Index, and VAS-GBD of tremor.The frequency/severity of AEs were evaluated primarily by the blinded rater, supplemented by observations by the unblinded physician responsible for stimulator programming.

### Localization of active contacts

To localize leads and active contacts, preoperative MRI and post-surgical volume CT scans (obtained median 155 days (10th-90th percentile 7–479) post-surgery were merged using SureTune software v3 (Medtronic^®^). Lead localizations were also linearly normalized to MNI-space (ICBM 2009b NLIN asymmetric) based on the local Yelnik atlas registration (Yelnik et al., 2007) using an in-house MATLAB toolbox (https://github.com/visualDBSlab/ARENA) that also utilized the Lead-DBS toolkit. The localizations of the active contacts at one-year follow-up were thus evaluated both with individual AC-PC x, y,z-coordinates and in common MNI-space.

The dentato-rubro-thalamic tract (DRTT) was delineated using the Petersen connectome. The estimated volume of tissue activated (VTAs) were calculated from individual chronic stimulation parameters for the active contact using SureTune, based on the Åström model. To evaluate the impact of the individual VTAs on the DRTT, we calculated the shortest distance between the VTA border/shell and the DRTT and performed individual analyses of number of fibers reached by the VTA. The distance from the center of gravity of individual activated contacts to the DRTT wasalso calculated.

### Statistical analysis

For power calculation and the statistical methods used to analyze primary endpoint, see [14]. Continuous baseline epidemiological and clinical data were described with median(minimum-maximum) whereas median(25th -75th percentile) was used for preoperative and one-year outcome scores, because the latter were not normally distributed. For secondary endpoints, we evaluated change of scores from preoperative to one-year follow-up using paired sample T-tests to calculate mean of paired (intra-individual) differences with 95% confidence interval (CI). Normal distribution for these paired differences was evaluated by histograms and Q-Q plots. A p-value < 0.05 was considered statistically significant.

Statistical analyses were performed using IBM SPSS Statistics for Windows 26.0 (IBM Corp, USA) and STATA/SE for Windows 16.1 (StataCorp LLC, USA).

## Results

### Study population

Between April 2015 and December 2019, double-blinded follow-up at one year post-surgery was performed in 43 patients (96%). Table 1 shows baseline demographics and clinical characteristics of the total population and the four diagnostic categories represented: ET, PD, dystonic tremor (DT) and cerebellar tremor (CbT).

**Table 1:** Baseline demographics and clinical characteristics

	Total *N* = 43	ET *N* = 16	PDT *N* = 10	DT *N* = 7	CBT *N* = 10
Gender					
Women	19 (44)	6 (37)	1 (10)	6 (86)	6 (60)
Men	24 (56)	10 (63)	9 (90)	1 (14)	4 (40)
Age at tremor onset (years)	35 (1–69)	28 (7–62)	62 (24–69)	35 (3–65)	31 (1–63)
Age at surgery (years)	68 (27–74)	62 (41–74)	72 (63–74)	70 (49–71)	62 (27–73)
Disease duration (years)	26 (2–68)	32 (7–50)	9 (2–39)	29 (5–68)	28 (5–53)
1st /2nd degree relative w/ tremor	28 (65)	14 (88)	2 (20)	6 (86)	6 (60)
Body distribution of tremor					
UL only	9 (100)	6 (67)	2 (22)	0 (0)	1 (11)
+ head/neck	4 (100)	2 (50)	0 (0)	1 (25)	1 (25)
+ head/neck + cranial	7 (100)	2 (29)	0 (0)	3 (43)	2 (29)
+ head/neck + truncal + LL	7 (100)	1 (14)	0 (0)	1 (14)	5 (72)
+ head/neck + LL	8 (100)	4 (50)	1 (13)	2 (25)	1 (13)
+ LL	7 (100)	1 (13)	7 (87)	0 (0)	0 (0)
Preop. DUL FTMTRS scores					
- Sum items 5/6 + 10–14 (0–32)	22 (8–32)	19 (8–30)	18 (13–32)	27 (10–29)	25 (10–30)
- Items 5/6 (0–12)	8 (4–12)	8 (5–12)	11 (6–12)	9 (4–11)	8 (4–10)
- Rest	2 (0–4)	1 (0–4)	4 (2–4)	2 (0–3)	1 (0–2)
- Postural	4 (1–4)	3 (1–4)	4 (2–4)	3 (1–4)	4 (2–4)
- Kinetic/intention	4 (1–4)	4 (2–4)	3 (1–4)	4 (2–4)	4 (2–4)
- Item 10	2 (0–4)	2 (0–4)	2 (0–4)	3 (1–4)	2 (1–4)
- Item 11	3 (0–4)	2 (1–4)	2 (0–4)	4 (1–4)	4 (1–4)

Data are median (minimum- maximum) for continuous variables and number (%) for categorical variables

ET = Essential tremor, PDT = Tremor in Parkinson´s disease, DT= Dystonic tremor, CbT= Cerebellar tremor, UL= Upper Limb, LL= Lower Limb, DUL= Dominant Upper Limb, FTMTRS= Fahn-Tolosa-Marin Tremor Rating Scale

Among the ET patients (*n* = 16), three had polyneuropathy mainly affecting the lower limbs, thus not regarded causal for the arm tremor. Among the PD patients (*n* = 10), two had been diagnosed and treated for ET before typical PD symptoms and DaTscan pathology were observed at age 66 and 55 years (age at tremor onset/surgery 61/69 years and 24/63 years, respectively). All DT patients (*n* = 7) had co-existing head tremor, and all but one familial tremor (affected 1st /2nd degree relatives). A 68 year-old man with familial, cervico-brachial DT starting at age 22, had thumb rest tremor and pathological DaTscan two years pre-surgery, but no bradykinesia, rigidity or levodopa-response on tremor preoperatively or the first postoperative year (although these appeared gradually with longer follow-up).

Among the CbT patients (*n* = 10), six had generalized and four segmental upper body tremor. In three patients tremor was considered secondary to advanced MS (disease duration 12–16 years, tremor onset 5–15 years preoperatively), with preoperative MRI´s showing known demyelinating white matter changes supratentorially in all, plus infratentorially and in the cervical spinal cord in two. Six patients had 1st /2nd degree relatives with idiopathic tremor, including two MS patients. In two patients with spinocerebellar ataxia (SCA) pathogenic sequence variants were found. In a man aged 27 years at surgery, who had learning difficulties, onset of tremor and ataxia during childhood and familial tremor with autosomal dominant pattern, a SCA21 mutation was found (TMEM240, c196 G > A, p.Gly66Arg) [21, 22]. In a man aged 31 at surgery, with tremor onset at age 15 and slowly progressive signs of a SCA, initial gene panels were negative, but re-testing showed mutations in the POLR3A gene (c.1909 + 22G > T and c.3655 G > T) [23, 24]. Two of the remaining four patients with idiopathic CbT (age at surgery 68–73) had familial tremor with onset in early adulthood and a history of alcohol abuse, one also reported narcotic drug abuse in early adulthood.

### Outcomes

The primary endpoint of the randomized phase showed significantly greater reduction of Sum dominant arm tremor score in the PSA-DBS period (mean paired differences VIM–PSA of -2.65, 95% CI -4.33 to -0.97, *p* = .002), corresponding to median improvement from preoperative of 88% with PSA-DBS and 80% with VIM-DBS, as previously reported [14]. Arm tremor disability and the patient-rated measures VAS-GBD tremor and QUEST physical domain score also improved significantly more in the PSA period [14].

At one-year follow-up, the median Sum dominant arm tremor score had improved from 24 to 4 (mean paired differences 15.7 (95% CI 13.8 to 17.7, *p*<.001) (Table 2). Significant improvements from preoperative to one-year follow-up were also found in all other secondary endpoints: FTMTRS Sum non-dominant arm tremor score, FTMTRS scores for all other body regions, total FTMTRS score, and patient-rated VAS-GBD of tremor and QUEST-SI (*p*<.001, Table 2).

**Table 2:** Secondary endpoints: Paired differences from preoperative to the blinded one-year follow-up

FTMTRS scores	Baseline		One year		Paired differences		
	*N*	Median (25th -75th *p*)	n*	Median (25th -75th *p*)	*n*	Mean (95% CI)	*P*
**Dominant arm**							
Item 5/6^*a*^ (0–12)	45	8 (7–10)	43	1 (0–3)	43	6.6 (5.8 to 7.4)	<0.001
Items 10-14^*b*^ (0–20)	45	13 (10–18)	43	3 (0–7)	43	9.1 (7.6 to 10.7)	< 0.001
Sum arm (0–32)	45	24 (17–27)	43	4 (0–9)	43	15.7 (13.8 to 17.7)	< 0.001
**Non-dominant arm**							
Item 5/6^*a*^ (0–12)	39	8 (7–9)	37	2 (0–4)	37	5.5 (4.6 to 6.4)	< 0.001
Items 11-14^*b*^ (0–16)	39	10 (8–15)	37	4 (1–6)	37	6.3 (4.8 to 7.9)	<0.001
Sum arm (0–28)	39	18 (14 − 24)	37	4 (2–9)	37	11.8 (9.8 to 13.8)	< 0.001
**Other body regions** ^*c*^							
Face/tongue/voice	32	3 (1–4)	30	0 (0–1)	30	2.4 (1.9 to 2.9)	< 0.001
Head/neck	26	2 (1–3)	24	0 (0–1)	24	1.3 (0.9 to 1.8)	< 0.001
Trunk	11	1 (1–2)	9	0 (0–0)	9	1.4 (0.7 to 2.2)	0.003
Dominant leg	40	2 (2–4)	39	0 (0–1)	39	2.5 (1.7 to 3.3)	< 0.001
Non-dominant leg	32	2 (1–3)	30	0 (0–1)	30	2.0 (1.5 to 2.6)	< 0.001
**Disability arms**							
Items 16-21^*d*^ (0–24)	45	17 (13–19)	43	4 (1–8)	43	10.5 (8.8 to 12.2)	<0.001
**Total score** (0-156)	45	64 (51–78)	43	12 (5–29)	43	43.4 (38.1 to 48.7)	<0.001
**QUEST SI** (0-100)	45	41 (32–52)	43	13 (3–25)	43	26.7 (22.1 to 31.2)	<0.001
**GBD Tremor** (0-100)	45	82 (74–94)	43	20 (9–56)	43	49.7 (40.4 to 58.9)	<0.001
**GBD Pain** (0-100)	45	13 (2–56)	43	1 (0–14)	43	15.5 (5.2 to 25.8)	0.004^#^

*Two complete drop outs that occurred late in the second randomized phase account for reduced n at the one-year follow-up. For non-dominant arm tremor *N* = 37 because six of ten PD patients were treated only unilaterally (all in their dominant (left) hemisphere for right-sided tremor).^#^Not significant after Bonferroni correction of alpha (0.0031)

FTMTRS (Fahn-Tolosa-Marin Tremor Rating Scale) contains 21 items: Item 1–14 score tremor severity in all body regions, from 0 = no tremor/disability to 4 = severe tremor (constant/marked amplitude/inability to perform a task due to tremor). ^*a*^ Items 5/6: sum of arm tremor (right/left) at rest+postural+kinetic. In this study, we included for the evaluation of postural tremor: arm stretched forward, wing-position and holding a filled paper cup. For kinetic tremor: both finger-to-nose, Barany´s test and drinking from a filled paper cup. ^*b*^ Item 10: Hand-writing (only scored for dominant arm); item 11–13: Drawing large spiral, small spiral, continuous lines, respectively; item 14, Pouring from one paper cup to the other. ^*c*^ Other body regions: Item 1–3: Face, tongue, voice, Item 4: Head/neck tremor (rest+posture), Item 7: Trunk (rest+posture), Item 8/9: sum of leg tremor (right/left) at rest+postural+kinetic. ^*d*^Item 16–21: Disability caused by arm tremor in daily activities (feeding, drinking, hygiene, dressing, writing and working) through patient interview (not pre-specified as secondary endpoint, but is shown for complete information). Abbreviations: CI – confidence interval; FTMTRS – Fahn-Tolosa-Marin Tremor Rating Scale; 25th-75th p - 25th-75th percentile; QUEST SI – Quality of Life in Essential Tremor Summary Index; GBD Tremor – Visual Analog Scale Global Burden of Disease

**Fig. 2:**
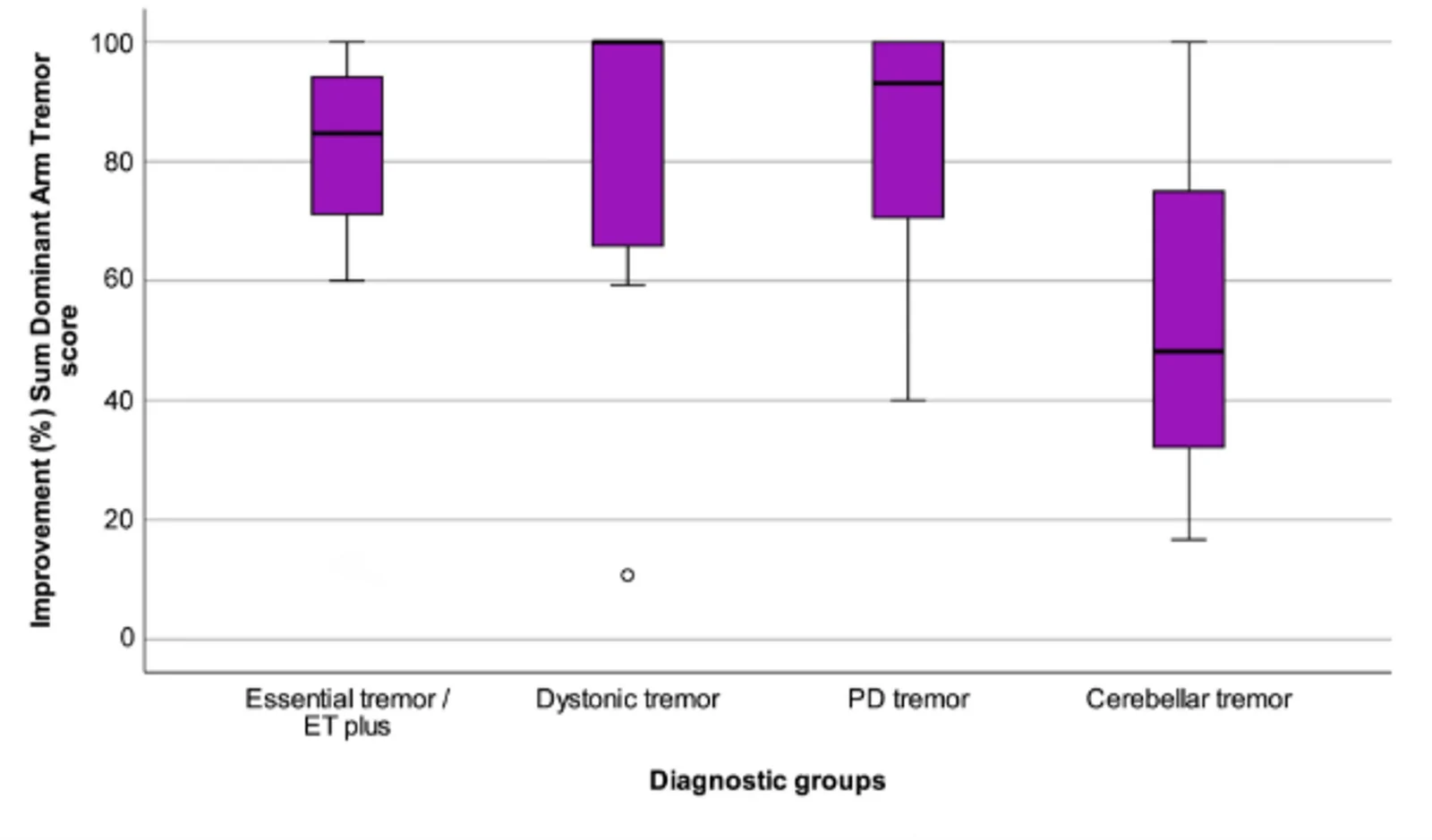
Relative (%) improvement from preoperative to the one-year follow-up of Sum Dominant Arm tremor, in the four diagnostic categories (Essential tremor, *n* = 16, Dystonic tremor, *n* = 7, Parkinson´s disease (PD) tremor, *n* = 10, Cerebellar tremor, *n* = 10)

**Fig. 3:**
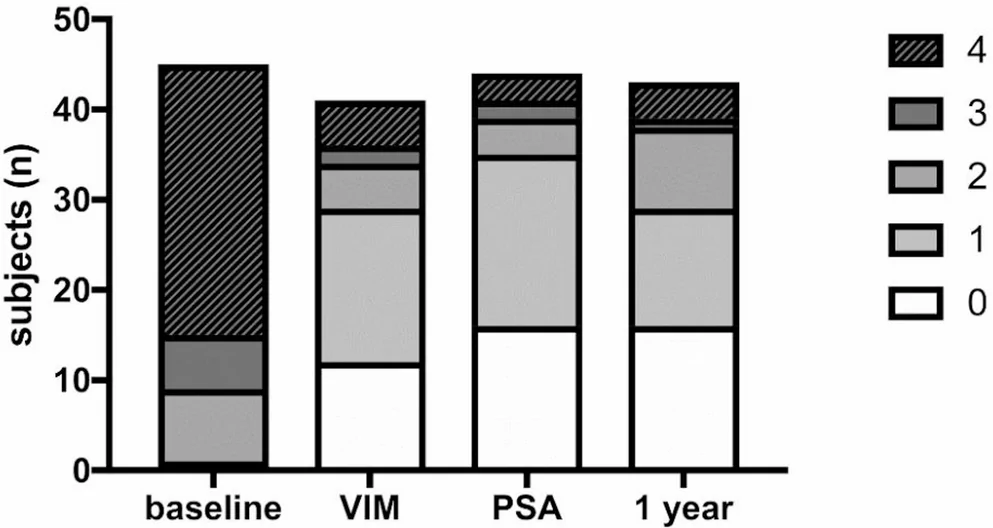
The proportion of patients with kinetic tremor score (0–4) at baseline compared to the randomized VIM- and PSA-DBS periods and the one-year follow-up The kinetic tremor score is part of the FTMTRS item 5/6 (right/left arm), and was in this study scored during active movement in the finger-to-nose test, Barany´s test, and bringing a water-filled plastic cup to the mouth and drink

Median percentage improvement of Sum dominant arm tremor was 79% in the total population, 85% in ET, 93% in PD, 100% in DT and 48% in CbT (Fig. 2). Median improvement of action tremor (postural + kinetic) was 83% for ET, 100% for PD and DT, and 55% for CbT, and for rest tremor 100% in all categories (Supplementary Table 1). Figure 3 shows the severity of kinetic tremor at baseline, at the end of the PSA-DBS versus the VIM-DBS randomized periods, and at one year. Median improvement of FTMTRS arm tremor disability score was 78% in the total population, about 90% in ET, PD and DT, but only 35% in the CbT patients (Supplementary Table 1). Significant improvements at one-year follow-up were also found for HADS Anxiety (*p*=.021) and Depression scores (*p*=.003) (*n* = 41), and in the minority of patients with scores above cut-off at baseline (HADS-A, *n* = 7, *p*=.018, HADS-D, *n* = 5, *p*=.043, Wilcoxon Signed Rank tests).

In the PD patients, UPDRS part III scores improved significantly, also for rigidity and bradykinesia in extremities and axial symptoms (Table 3).

**Table 3:** Improvement from baseline to the blinded one-year follow-up in UPDRS III scores (medication-off, stimulation-on) in the Parkinson´s disease group, *n* = 10

UPDRS items	Baseline Median (25th -75th perc.)	One year Median (25th -75th perc.)	Paired differences Mean (95% CI)	*P*
**Tremor** Right^*a*^ extremities	8 (8–10)	0 (0-1.3)	7.9 (6.3–9.5)	< 0.001
**Rigidity** Right^*a*^ extremities	3 (1.0-4.3)	0 (0–1)	2.3 (1.3–3.3)	< 0.001
**Bradykinesia** Right^*a*^ extremities	7 (5-8.5)	2.5 (1.8–5.5)	3.5 (2.0–5.0)	< 0.001
**Axial** symptoms	8 (6.3–9.3)	3.5 (1.8-5.0)	4.6 (2.2-7.0)	0.002

^*a*^Only scores for right extremities + midline are shown because six of ten patients received only unilateral DBS in left (dominant) hemisphere. However, in the four PD patients who received bilateral stimulation, similar baseline scores and improvements were observed in left extremities, with paired mean differences: tremor 7.8, rigidity 2.5 and bradykinesia 4.5 (*p*=.001,0.003,0.003, respectively)

UPDRS III=Unified Parkinson’s Disease rating Scale, part III (motor examination). CI=Confidence Interval

### Post hoc analyses of changes from 6 to 12 months in active contacts and tremor scores

In randomization group 1 (VIM-DBS 1–3 months, PSA-DBS 4–6 months, *n* = 22), the active contact in dominant hemisphere was changed in only one patient (level 0 to 2). Improvement of Sum dominant arm tremor score remained stable in this and 11 other patients (no change or +/-1 point), improved further in two (by 2–5 points), and worsened in eight (median 6 points, range 2–10). In randomization group 2 (PSA-DBS 1–3 months, VIM-DBS 4–6 months, *n* = 21), two patients were already stimulated in PSA at the six-month follow-up due to intolerable adverse effects from VIM-DBS. In 16 patients, the active contact was changed to one of the two most ventral, resulting in further improvement of Sum dominant arm tremor score in seven patients (median 4 points, range 4–10), a stable effect in seven, and worsening in two (2–6 points). In three patients with unchanged active contact the score worsened by 3–6 points.

Taken together, of the 41 patients with valid sum dominant arm tremor score at the six-month follow-up, nine (22%) experienced further improvement at one year, 19 (46%) had stable effect, and 13 (32%) experienced worsening. Worsening was mainly observed in the spiral/line drawing scores, whereas both item 5/6 and handwriting scores remained stable. The frequency of worsening in sum scores from 6 to 12 month follow-up was not significantly different between the two randomization groups, and was 56% in the CbT subgroup, 43% in DT, 31% in ET and 0% in PD (*p* = .041, Fisher-Freeman-Halton Exact Test). No patient experienced therapy escape of tremor suppression (return to baseline scores) in the dominant arm. In a PD patient with bilateral DBS for very severe rest and action tremor bibrachially, therapy escape was observed in the non-dominant arm for the action tremor component only. Two patients with CbT (and previous alcohol abuse) had some habituation tendency (one bilaterally, one non-dominant arm).

### Adverse events

Adverse events (AE) observed 6–12 months postoperatively that could be related to the intervention are shown in Table 4. AE were more frequent in patients with combined tremor types than in ET patients of whom 50% had no AE. The most common AE was a subjective sense of disequilibrium without any objective findings (*n* = 10). Three patients had impaired tandem gait only, seven moderate gait ataxia or a fall (16%), and none had severe gait ataxia.

**Table 4:** Type of Adverse Events (AEs) possibly related to treatment intervention (DBS) during six months to one year postoperative

	Total	Diagnostic category			
	*N* = 43	ET *N* = 16	PD *N* = 10	CBT *N* = 10	DT *N* = 7
**No Adverse Event**	**17 (40)**	**8 (50)**	**4 (40)**	**3 (30)**	**2 (29)**
**Gait/Balance**	**20 (47)**	**6**	**6**	**5**	**3**
Subjective disequilibrium	10	4	3	1	2
Impaired tandem gait only	3	2	1	0	0
Moderate gait ataxia or fall	7	0	2	4	1
Severe gait ataxia	0	0	0	0	0
Speech/voice*	**7 (16)**	**1**	**3**	**1**	**2**
Subjective dysarthria	3	1	0	1	1
Objective dysarthria	2	0	2	0	0
Hypophonia/ hoarse voice	2	0	1	0	1
**Motor**,** other**	**1 (2)**	**0**	**0**	**0**	**1**
Dysphagia	0	0	0	0	0
Reduced dexterity (contralateral)	0	0	0	0	0
Dyskinetic episode	1	0	0	0	1
**Somatosensory disturbances (contralateral)**	**2 (5)**	**1**	**0**	**0**	**1**
Paresthesias	2	1	0	0	1
Numbness	0	0	0	0	0
**Dysgeusia**	**2 (5)**	**0**	**1**	**1**	**0**

Numbers are n (%). Bold font highlights the major categories of adverse events and the number of such events in each diagnostic category. Five patients had more than one adverse event: One ET patient had subjective disequilibrium and subjective dysarthria, one PD patient had a fall and dysgeusia, another PD patient had a fall and hypophonia. One patient with dystonic tremor had subjective disequilibrium and paresthesias, and another patient with dystonic tremor had subjective dysarthria and hoarse voice. The latter patient had a preoperatively a pathological DATscan, but no parkinonsim and no levodopa response to his dystonic tremor, but with follow-up beyond one year Parkinson´s disease was diagnosed

Comorbidity with polyneuropathy of lower extremities may have contributed to subjective disequilibrium in three ET patients and to a fall in a PD patient. Other common comorbidities that may have contributed to balance problems and falls were preoperative cerebellar ataxia, joint disorders, and chronically increased alcohol consumption. Thus, DBS may have contributed, but was unlikely the only cause of balance problems at one-year follow-up. Objective dysarthria was only observed in two patients with PD. Dysgeusia was observed in two patients, but was resolved with stimulator adjustments. One patient reported a very brief, self-limiting episode of dyskinesia.

### Stimulation parameters and localization of active contacts

At one-year follow-up, mean(SD) stimulation parameters in the dominant/non-dominant hemisphere (*n* = 43/37) were: current 2.4(0.8)/2.2(0.7) mA, pulse width 60(0)/60(0) microseconds, frequency 135(14)/136(14) Hz. In the group with PSA-DBS months 4–6, mean(SD) current was increased from 2.1(0.7) at six-month to 2.3 (0.8) at one-year follow-up and from 2.0(0.6) to 2.2(0.7) in the left and right hemispheres, respectively. In the group with VIM-DBS months 4–6, mean(SD) current was reduced from 2.6(0.5) to 2.3(0.6) and from 2.4 (0.6) to 2.2 (0.6) in the left and right hemispheres, respectively. In the five leads with active contact localized above the AC-PC plane, stimulation currents were 1.2, 2.0, 2.5, 2.8 and 3.5 mA.

**Fig. 4:**
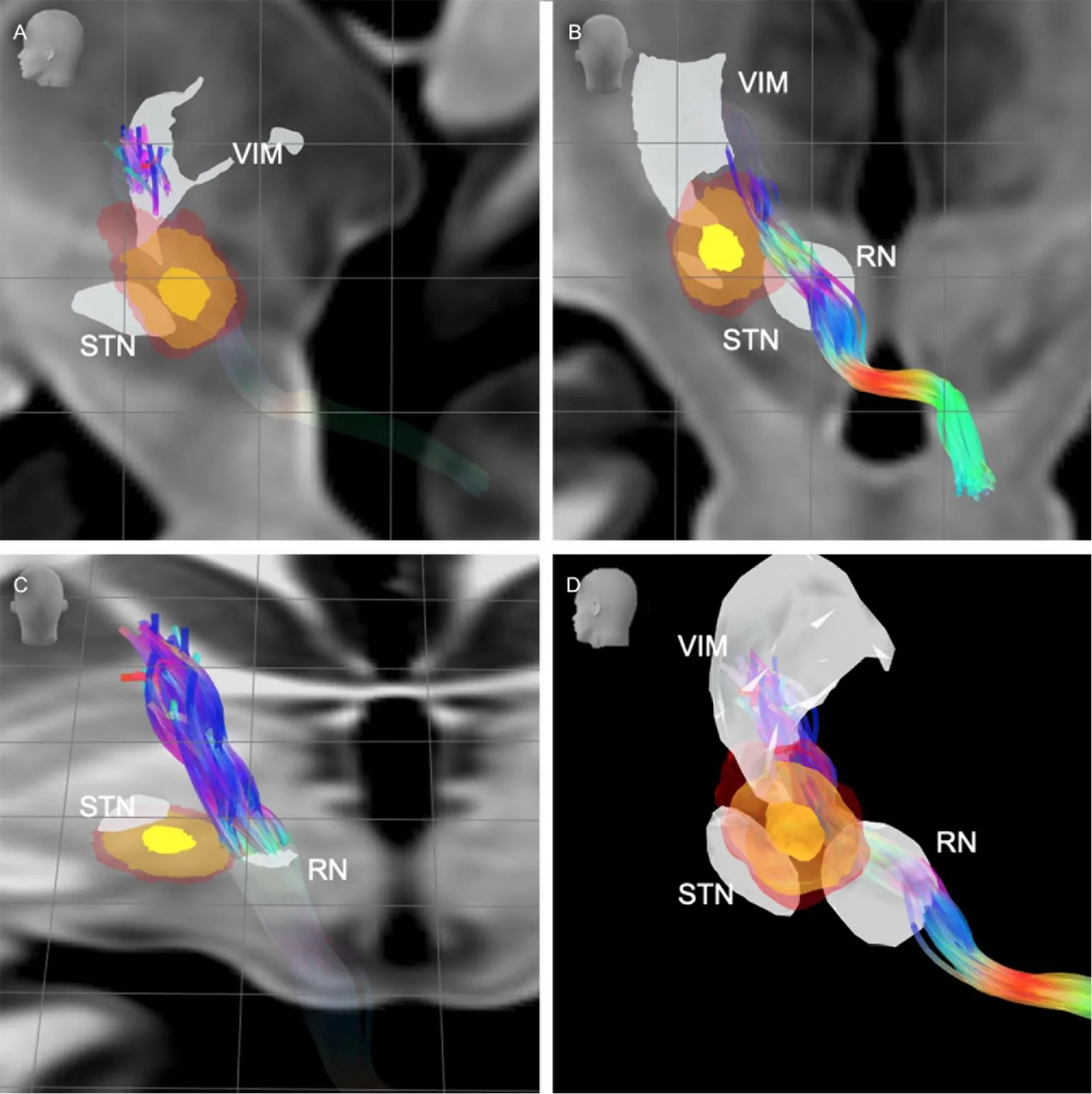
The localization of the active contacts stimulated at the one-year follow-up, displaying their estimated Volume of Tissue Activated (VTA). The aggregated stimulated area is shown in yellow-orange-red in the sagittal (**A**), coronal (**B**) and axial (**C**) planes, and in a 3D depiction (**D**), on the anatomy of the ventro-lateral thalamic and posterior subthalamic area (PSA). The ventral intermediate thalamic nucleus (VIM), subthalamic nucleus (STN) and nucleus ruber (RN) are depicted together with the dentato-rubro-thalamic tract (; the fiber tract shown). Not depicted is the Zona Incerta nucleus, which is localized in the PSA target area (see ref.11). The colors of the clusters refer to the Gaussian Sigma, with 1 Sigma = 64% of the data being inside the yellow core ( 26 VTAs or more), 2 sigma = approx 95% being inside the orange field (4 VTAs or more), and 3 sigma = approx 99% = being inside the red area (1 VTA or more). The mean(SD) AC-PC based coordinates of the active contacts in the dominant/non-dominant hemispheres were: X (mm lateral to the inter-comissural line) = 12.40(1.23)/11.91(1.06), Y (mm anterior-posterior relative to MCP) = − 5.81(1.71)/-6.02(1.42), and Z (mm superior/inferior to the AC-PC plane) = − 3.52 (1.64)/-3.64(1.43). The mean(SD) MNI based coordinates in the dominant/non-dominant hemispheres were X = − 12.40(1.23)/ 11.91(1.06), Y= -17.55(1.76)/ -17.77(1.48), and Z= -5.79 (1.94)/-5.95 (1.67). Grid: 1 cm. The AC-PC based Z-coordinates were below the AC-PC-plane (thus subthalamic) in all but five active contacts: three in the dominant (+ 0.7,+1.0,+ 2.2 mm) and two in the non-dominant (+ 0.53, + 0.54 mm) hemisphere. For all of these, however, the VTA overlapped with the DRTT

Figure 4 shows the localization of the VTA by chronic stimulation of the patients (*n* = 42/36 in dominant/non-dominant hemisphere). The mean(SD) AC-PC- and MNI-based coordinates for the center of gravity of the active contacts´ in both hemispheres are referred in the legend. The z-coordinate was below the AC-PC-plane in 94% (73/78).

The distance between the individual VTA shell and the dentato-rubro-thalamic tract (DRTT) was calculated as zero in 36/78 hemispheres (46%); in 18/42 (43%) dominant hemispheres and in 18/36 (50%) non-dominant hemispheres. In these patients, excellent improvement (≥70%) of Sum arm tremor score was observed in 15/18 (83%) dominant arms and in 9/18 (50%) non-dominant arms. In the remaining patients, with median distance from individual VTA shell to the DRTT of 1.02 mm (range 0.08–2.78), excellent improvement (> 70%) was observed for dominant arm tremor in 16/24 (67%) patients and for non-dominant arm tremor in 12/18 (67%). Individual fiber analysis showed that the VTA of 40/78 (51%) active contacts reached the DRTT; 19/42 (45%) in dominant and 21/36 (58%) in non-dominant hemisphere, with median *n* = 7 fibers (both hemispheres, range 1-437 /1-279). Spearman Rho correlation coefficients between n DRTT fibers reached by the VTA and sum arm tremor improvement were 0.370 (*p*=.119)/ 0.068 (*p*=.769), for the dominant/non-dominant hemisphere/arm. Improvement of contralateral arm tremor was not significantly different between the subgroup of patients with the DRTT reached and the subgroup without (Mann Whitney U-test, *p*=.574/*p*=.511, for dominant/non-dominant arm). The distance from the VTA shell to the DRTT strongly correlated with the distance from the active contact´s center of gravity to the DRTT (Spearman´s rho 0.926, p = < 0.001).

The VTA overlap with the STN volume was median 7.5% (25,75 percentile: 0,21) in the dominant hemisphere and 3% (0,19) in the non-dominant hemisphere, and in the PD-group this overlap was 7% (1,19) in the dominant and 0% (0,0) in the non-dominant hemisphere. No significant correlations were found between the percentage overlap of the STN by the VTA in MNI space and the improvement of contralateral UPDRS scores for tremor, rigidity, bradykinesia or axial symptoms.

## Discussion

We have presented the outcomes of the double-blinded one-year follow-up of DBS on tremor suppression, arm function and quality of life in 43 patients with action tremor as part of the most common tremor disorders. This constituted the predefined secondary endpoints of our randomized controlled crossover study, which showed that PSA-DBS was superior when compared with VIM-DBS for three months each during the first six months post-surgery [14]. After the six-month randomized phase, individual optimization of stimulation parameters through clinical testing, including free change of active contact, resulted in change to one of the two most ventral contacts in 84% of those treated with VIM-DBS during months 4–6 post-surgery and only one change to a more dorsal contact in those treated with PSA-DBS. At one year post-surgery, all secondary endpoints (evaluated as paired differences from preoperative to the one-year follow-up) were significantly improved: the objective double-blinded ratings of tremor severity in all body parts, functional disability of arm tremor, and the patient-rated measures VAS-GBD of Tremor and QUEST-SI. Median improvement of the Sum Dominant Arm tremor score versus preoperative was 79% in the total population, 85% in ET, 93% in PD, 100% in DT and 48% in CbT. Disability of arm tremor improved by median 78% in the total population, around 90% in ET, PD and DT, but only 35% in the CbT patients. From the six-month to the one-year follow-up, two-thirds of patients either had a stable improvement (46%) or improved further (22%). One-third experienced some worsening of tremor scores, most frequently in the CbT patients and in the spiral and line drawing items.

Imaging evaluation of the individual active contact/VTA localization at the one-year follow-up showed that 94% were in the PSA, while 6% were slightly above the AC-PC plane. In MNI space, the calculated distance from the VTA shell to the DRTT was zero for 46% of the active contacts (including those localized just above the AC-PC plane) and median 1 mm for the remaining VTAs. Thus, our results at one-year follow-up can be viewed as a report of PSA-stimulation, including the Zona Incerta [11]. The DRTT was stimulated in a subgroup of patients.

Our patient cohort was clinically heterogenic, but all had troublesome action tremor as the main inclusion criterion. The outcomes in the total study population may therefore not be directly comparable with reports of pure ET or PD cohorts treated with PSA-stimulation. Our results in the ET, PD and DT groups are, however, all very good. Both objective tremor suppression and improvement of arm function are in the upper range compared to other publications reporting one-year follow-up, many of which were non-blinded, as reviewed by Kuebler et al. [8]. In their own cohort of 30 ET patients, they reported an average 57% improvement on blinded video-evaluations, using a similar upper limb tremor score as we did. They found that maximum average tremor improvement was 70.5% in a voxel with MNI coordinates x=-12, y=-19.5 and z=-5.5 [8], similar to the mean MNI-coordinates of our VTAs at the one-year follow-up. In a randomized controlled crossover study similar in design to ours, with 13 ET patients included, Barbe et al. found a non-significant trend (*p* = .086) for better arm tremor suppression in the two-month PSA-period (68%) versus the two-month VIM-period (54%) [25]. They revisited these results by calculating in MNI space the distance from the DRTT to the contacts in VIM and PSA, respectively, and found that the contacts in the PSA were more “efficient” (tremor improvement after two months of DBS/mA stimulated) and localized significantly closer to the DRTT [26]. A double-blind randomized study of 34 patients with ET or tremor-dominant PD that compared PSA targeting by conventional landmarks to targeting directly on the DRTT, reported that in the latter group the electrodes were closer to the DRTT and provided better improvement of arm tremor and function, from six months to 60 months postoperative, with lower stimulation amplitudes and pulse widths [27]. Together these studies provide emerging evidence that the PSA is a very good target for tremor suppression in ET, but also for tremordominant PD, and that reaching the DRTT with the VTA may be important for more optimal and longer lasting effects [27]. In our study, excellent improvement of dominant arm tremor was observed even more often if the distance between the VTA shell and DRTT was zero, but was also observed with distances > 0 (median = 1 mm) in most ET, PD or DT patients. For non-dominant arm tremor similar good effects in ET, PD and DT were observed both when the VTA reached the DRTT and when lying close to it. Here it must be underlined that using MNI space to evaluate the overlap of individual VTA and DRTT may represent a methodological limitation to our study (and other studies referring to DRTT evaluated in MNI space only), because the patients´ individual anatomy may not be reflected with millimeter accuracy.

The fact that PSA stimulation can provide good tremor suppression even without stimulating the DRTT can probably partly be explained by stimulation of other passing tracts, such as the pallido-thalamic tract (PTT). The PTT has been shown to be involved in the suppression of dystonic tremor by thalamic DBS, but not of ET [28]. In an unblinded series of MRI guided focused ultrasound lesions directed to the PTT a good effect on PD tremor was reported [29].

Some recent studies have reported data indicating that the effects of DRTT stimulation the may depend on whether the VTA overlap the crossing and major part of the tract, as opposed to the smaller non-crossing part. At one month post-surgery, Deuter et al. found that contacts close to the crossing fibers better improved intention tremor, while postural tremor was suppressed by both crossing and non-crossing fiber stimulation [30]. In VIM-DBS for ET both the crossing and the non-crossing fibers contributed to a mean contralateral tremor reduction of 62.5%, with follow-up of 6.6(1.8) months [28]. In a recent study, Sajonz et al. focused on ET patients with long-term treatment (mean 5.6 years, maximum16), and reported that for the VTAs in the non-dominant hemisphere a disproportional stimulation of the non-crossing versus the crossing fibers was associated with both a higher risk for delayed therapy escape (;similar or worse tremor than preoperatively, *n* = 5/29) and for development of ataxia [31]. They suggest that this disadvantageous effect of a disproportionate stimulation of the uncrossed fibers probably is only relevant with long-term treatment (> 12 months).

In our study, tremor suppression at one-year follow-up was similar for dominant and non-dominant arm, except in the drawing tasks for which it seems natural that more tremor is provoked when using the non-dominant hand. We unfortunately have no data on the ratio of stimulation of crossed versus uncrossed DRTT fibers in our patients. The main predictor for less good tremor suppression after one-year of DBS was in our study population to have a cerebellar tremor phenotype preoperatively, as this group had less percentage reduction of tremor scores and arm function improvement on average compared to ET, PD and DT, and a higher frequency of deterioration from the six-months to the one-year follow-up. However, high-amplitude wing-beating postural tremor and intention tremor were generally the most difficult tremor types to suppress in a complete and sustained way, also in two PD patients who had little levodopa-response to such tremor and severe rest tremor.

In the PD group in general we observed very good effects on rest tremor and postural tremor with arm stretched forward, and also on bradykinesia and rigidity, while more moderate response was seen on high-amplitude wing-beating postural and intention tremor. Levodopa-resistant tremor in PD has been shown to be related to more tremor-amplitude related activity in cerebellar cortex and nuclei, in particular the interposed nuclei, so levodopa-resistance together with a more cerebellar type tremor in PD patients may represent a subgroup who have more cerebellar pathology [32]. The observed improvement in rigidity and bradykinesia in our PD subgroup may be explained by modulation of fiber pathways passing through the posterior subthalamic area, including pallido-thalamic projections. However, as tract-specific stimulation of pallido-thalamic fibers was not directly assessed in the present study, this interpretation remains speculative. Also, clinical assessment of rigidity and bradykinesia in tremor-dominant patients may be influenced by tremor severity, which we acknowledge could contribute to our findings.

Adverse events were generally mild, and moderate gait ataxia or fall was observed in seven patients, all with combined tremor syndromes (PD = 2, CbT = 4, DT = 1) and bilateral DBS in 6/7. Dysarthria was only seen in two PD patients. Dysarthria was in the randomized phase more frequent in the VIM-period than the PSA-period [14]. Treatment escape was only seen in the action tremor component in the non-dominant arm of one PD-patient with very severe preoperative bibrachial rest and action tremor.

To fully delineate the effect of PSA-DBS on the various tremor disorders, documentation of long-term effect beyond one year is needed and is ongoing in our population. This should be supplemented by larger cohorts of each tremor category in future studies [33].

## Supplementary Information

Supplementary material 1.

## Data Availability

All data supporting the findings of this study are available within the paper and its Supplementary Information.
