# Publisher Correction: Deep brain stimulation for arm tremor: one-year blinded follow-up of a randomized trial

**DOI:** 10.1186/s42466-026-00549-6

**Published:** 2026-11-05

**Authors:** Nadja Kvernmo, Martin M. Reich, Jonas Roothans, Ane E. Konglund, Are H. Pripp, Espen Dietrichs, Jens Volkmann, Inger Marie Skogseid

**Affiliations:** 1 Department of Neurology, Oslo University Hospital, PO box 4950, Nydalen, 0372 Oslo Norway; 2 Institute of Clinical Medicine, University of Oslo, Oslo, Norway; 3 Department of Neurology, University Hospital of Würzburg, Würzburg, Germany; 4 Visual DBS lab, University Hospital of Würzburg, Würzburg, Germany; 5 Department of Neurosurgery, Oslo University Hospital, Oslo, Norway; 6 Department of Biostatistics, Oslo University Hospital, Oslo, Norway; 7 Institute of Clinical Medicine, University of Würzburg, Würzburg, Germany

**Publisher Correction: Neurological Research and Practice (2026) 8:82**

https://doi.org/10.1186/s42466-026-00537-w

Due to a typesetting mistake, the following sentence, which is part of the main text, was inadvertently added also as part of the caption for Table 3 and has now been removed. “Significant improvements at one-year follow-up were also found for HADS Anxiety (p=.021) and Depression scores (p=.003) (n = 41), and in the minority of patients with scores above cut-off at baseline (HADS-A, n = 7, p=.043, HADS-D, n = 5, p=.043, Wilcoxon Signed Rank tests).”

The original article has been corrected.

