# Supplementary material for: Deep brain stimulation for arm tremor: one-year blinded follow-up of a randomized trial
Source: Neurol Res Pract. 2026 Oct 9;8(1):82. doi: 10.1186/s42466-026-00537-w (PMC13651327; doi:10.1186/s42466-026-00537-w)
Supplement: Supplementary file 1 [file 42466_2026_537_MOESM1_ESM.docx]

**Supplementary table 1. Relative improvement (in %) of tremor scores from baseline to one year follow-up, in the total population and the four diagnostic groups**

| **FTMTRS Dominant arm** | **Total**  n=43 % | **ET**  n=16 % | **PD**  n=10 % | **DT**  n=7 % | **CBT**  n=10 % |
| --- | --- | --- | --- | --- | --- |
| FTMTRS Sum Arm score *^a^* | 43 79 (59-100) | 16 85 (71-94) | 10 93 (66-100) | 7 100 (59-100) | 10 48 (30-76) |
| FTMTRS Item 5/6 *^b^* | 43 83 (67-100) | 16 83 (76-89) | 10 95 (71-100) | 7 100 (67-100) | 10 62 (48-80) |
| - *Rest* | 31 100(100-100) | 9 100(100-100) | 10 100 (94-100) | 6 100 (100-100) | 6 100 (100-100) |
| - *Action^c^* | 43 83 (60-100) | 16 83 (75-88) | 10 100 (66-100) | 7 100 (57-100) | 10 55 (34-78) |
| FTMTRS Item 10*^d^* | 40* 100 (75-100) | 14 100 (94-100) | 9 100 (13-100) | 7 100 (75-100) | 10 100 (56-100) |
| FTMTRS Items 11-13*^e^* | 43 79 (50-100) | 16 85 (53-100) | 10 100 (81-100) | 7 100 (58-100) | 10 43 (27-67) |
| FTMTRS Item 14*^f^* | 43 75 (33-100) | 16 100 (69-100) | 10 75 (0-100) | 7 100 (0-100) | 10 50 (0-81) |
| **FTMTRS Non-dominant arm** |  |  |  |  |  |
| FTMTRS Sum Arm score | 37 73 (55-86) | 16 76 (66-93) | 4 68 | 7 67 (39-88) | 10 60 (28-78) |
| FTMTRS Item 5/6 | 37 80 (58-100) | 16 85 (64-100) | 4 92 | 7 63 (50-80) | 10 58 (40-93) |
| - *Rest* | 25 100 (100-100) | 9 100 (100-100) | 4 100 | 5 100 (25-100) | 7 100 (100-100) |
| - *Action* | 37 75 (50-100) | 16 83 (60-100) | 4 88 | 7 60 (50-88) | 10 50 (29-91) |
| FTMTRS 11-13 | 37 63 (48-91) | 16 78 (58-100) | 4 32 | 7 83 (50-100) | 10 54 (30-73) |
| FTMTRS 14 | 37 67 (0-100) | 16 100 (50-100) | 4 0 | 7 50 (0-100) | 10 50 (0-81) |
| **FTMTRS 16-21 *Disability*** | 43 78 (43-95) | 16 90 (74-99) | 10 89 (54-97) | 7 94 (21-100) | 10 35 (13-71) |
| **FTMTRS Total** -bilat. DBS  -unilat DBS | 37 76 (56-90)  43 81 (60-94) | 16 83 (74-90)  0 | 4 70 (27-88)  6 91 (70-96) | 7 90 (39-95)  0 | 10 54 (38-77)  0 |
| **VAS-GBD *Tremor*** | 43 75 (25-88) | 16 88 (80-95) | 10 41 (17-76) | 7 84 (-2- 87) | 10 43 (12-73) |

Percent improvement is referred as median (25^th^ – 75^th^ quartile). Only median is shown if n<5. *^a^* FTMTRS Sum Arm score represent the sum of scores for items 5/6+10-14 for dominant arm and 5/6+11-14 for non-dominant arm. *^b^* Items 5/6: Sum arm tremor at rest + postural + kinetic. *^c^* Action tremor = Postural + kinetic tremor score. *^d^*Item 10: Hand-writing (dominant arm). *N=40 because two patients with ET and one with PD had normal handwriting both at baseline and at the one-year follow-up. *^e^*Items 11-13: Drawing large spiral (11), small spiral (12), continuous lines (13). Item 14: Pouring from one cup to the other.

Abbreviations: CbT – Cerebellar tremor, DT – Dystonic tremor, ET – Essential tremor, FTMTRS – Fahn-Tolosa-Marin Tremor Rating Scale, PDT – Tremor in Parkinson´s disease, VAS-GBD – Visual Analog Scale – Global Burden of Disease
